# Intestinal microbiota links to allograft stability after lung transplantation: a prospective cohort study

**DOI:** 10.1038/s41392-023-01515-3

**Published:** 2023-09-01

**Authors:** Junqi Wu, Chongwu Li, Peigen Gao, Chenhong Zhang, Pei Zhang, Lei Zhang, Chenyang Dai, Kunpeng Zhang, Bowen Shi, Mengyang Liu, Junmeng Zheng, Bo Pan, Zhan Chen, Chao Zhang, Wanqing Liao, Weihua Pan, Wenjie Fang, Chang Chen

**Affiliations:** 1 Department of Thoracic Surgery, Shanghai Pulmonary Hospital, School of Medicine, Tongji University, Shanghai, China; 2 Shanghai Engineering Research Center of Lung Transplantation, Shanghai, China; 3 State Key Laboratory of Microbial Metabolism, School of Life Sciences and Biotechnology, Shanghai Jiao Tong University, Shanghai, China; 4 Department of Thoracic Surgery, Changhai Hospital, Naval Medical University, Shanghai, China; 5 Department of Thoracic Surgery, The First Affiliated Hospital of Guangzhou Medical University, Guangzhou, China; 6 Department of Cardiovascular Surgery, Sun Yat-sen Memorial Hospital, Sun Yat-sen University, Guangzhou, China; 7 Department of Dermatology, Shanghai Key Laboratory of Molecular Medical Mycology, Shanghai Changzheng Hospital, Naval Medical University, Shanghai, China; 8 Adfontes (Shanghai) Bio-technology Co., Ltd, Shanghai, China

**Keywords:** Microbiology, Respiratory tract diseases, Translational research

## Abstract

Whether the alternated microbiota in the gut contribute to the risk of allograft rejection (AR) and pulmonary infection (PI) in the setting of lung transplant recipients (LTRs) remains unexplored. A prospective multicenter cohort of LTRs was identified in the four lung transplant centers. Paired fecal and serum specimens were collected and divided into AR, PI, and event-free (EF) groups according to the diagnosis at sampling. Fecal samples were determined by metagenomic sequencing. And metabolites and cytokines were detected in the paired serum to analyze the potential effect of the altered microbiota community. In total, we analyzed 146 paired samples (AR = 25, PI = 43, and EF = 78). Notably, we found that the gut microbiome of AR followed a major depletion pattern with decreased 487 species and compositional diversity. Further multi-omics analysis showed depleted serum metabolites and increased inflammatory cytokines in AR and PI. *Bacteroides uniformis*, which declined in AR (2.4% vs 0.6%) and was negatively associated with serum IL-1β and IL-12, was identified as a driven specie in the network of gut microbiome of EF. Functionally, the EF specimens were abundant in probiotics related to mannose and cationic antimicrobial peptide metabolism. Furthermore, a support-vector machine classifier based on microbiome, metabolome, and clinical parameters highly predicted AR (AUPRC = 0.801) and PI (AUPRC = 0.855), whereby the microbiome dataset showed a particularly high diagnostic power. In conclusion, a disruptive gut microbiota showed a significant association with allograft rejection and infection and with systemic cytokines and metabolites in LTRs.

## Introduction

Lung transplantation is a potentially curative therapy for patients with end-stage pulmonary disease.^1^ Nevertheless, the overall survival after a lung transplant is still inferior compared to other solid-organ transplantation modalities.^2,3^ For adult lung transplant recipients (LTRs) who survived 1 year after transplantation, the median survival increases from 6.7 to 8.9 years. Severe allograft rejection (AR) and pulmonary infection (PI) are the most common complications within 1 year after the transplant.^4^ These diseases are not only the major causes of death but are also associated with chronic lung allograft dysfunction (CLAD).^5^ Inflammatory allograft events, such as primary graft dysfunction, are associated with the subsequent development of AR and PI.^6–8^ Nevertheless, predispositions to the susceptibility to pulmonary rejection and infection are not fully understood.^2^

Previous longitudinal studies based on gene sequencing have revealed that the microbiome is appreciable in healthy subjects, altered in pathological diseases, and significantly associated with clinical outcomes.^9,10^ The reduced gut microbial diversity is correlates with allograft disease etiology and severity.^11,12^ Compelling evidences have also showed that the gut microbiome could modulate alloimmunity and rejection, directly implicating the gut microbiome as a therapeutic target in organ transplantation.^13,14^ In addition, longitudinal studies from patients undergoing liver and kidney transplantation have demonstrated disruption of the gut microbiome after transplantation was characterized by loss of diversity with important metabolic pathways and domination by a single species, and an increase in the prevalence of antibiotic resistance genes.^12^ These results supported that potential gut microbiome-targeted interventions could influence the survival of patients received solid organ transplantation.

The possibility that the microbiota of the lower respiratory tract may have local effects following lung transplantation has been widely reported.^15–17^ According to these studies, increased lower respiratory tract bacterial burden and lower diversity are associated with increased AR and inferior survival. Meanwhile, gut-associated bacteria were significantly enriched in the patents with pulmonary inflammation. More importantly, recent studies have shown intestinal microbiota is vital in determining respiratory diseases, such as asthma and atopy development.^18^ Interestingly, Wu et al. indicated that direct immune-mediating functions of the gut microbiota in AR mouse models occur via impairing Th17 responses.^19^ To date, whether the gut microbiome can be linked to allograft diseases in the setting of lung transplantation is currently unknown.

We hypothesized that the gut microbiota is associated with pulmonary disorders, including AR and PI, within 1 year after the transplant. A prospective multi-centers cohort study of LTRs undergoing per-protocol surveillance bronchoscopy was initiated. The association between the microbiota community composition in feces with the development of pulmonary diseases was evaluated. Subsequently, metabolites and cytokines from paired peripheral blood were examined to account for the microbial differences. Finally, we accurately diagnosed AR and PI from our multi-omics data using a machine learning approach.

## Results

### Participant and sample profiles

**Fig. 1:**
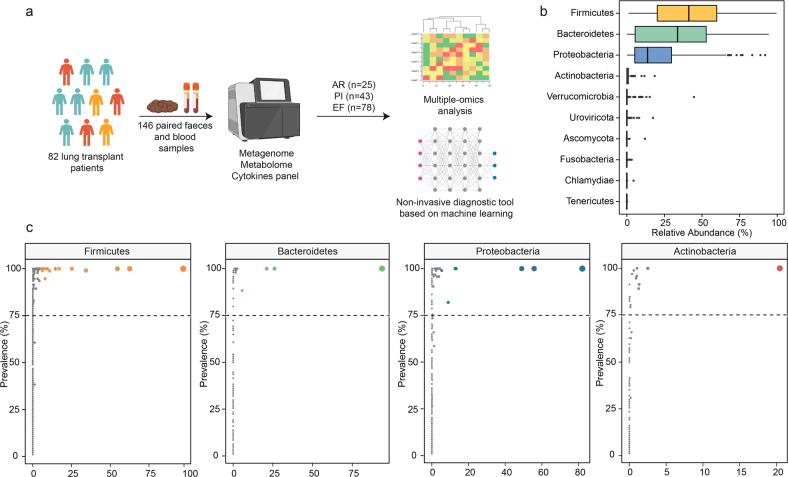
**a** Overview of the study design. The illustration was created with BioRender.com. **b** Relative abundances (%) of most abundant phyla across all fecal samples. Box plots show median (middle line), 25th, 75th percentile (box) and 5th and 95th percentile (whiskers) as well as outliers (single points). **c** Prevalence (% samples) of genus across samples for the four most abundant phyla. The *Y*-coordinate represents the prevalence of the genus in all samples and the *X*-coordinate represents the maximum relative abundance of the genus in a single sample. Dot color shows different genera and size show total rarefied reads

This multi-center prospective study involved 82 LTRs who underwent lung transplantation between May 2020 and December 2022 and provided the demographic characteristics in Supplementary Table 1. Over half of the pretransplant diagnoses were interstitial lung disease (51.2%). Furthermore, 146 pairs of feces and peripheral serum samples were analyzed after quality control (Fig. 1a). These samples were classified into three groups according to the diagnosis at the time of sampling (Supplementary Fig. 1). AR, PI and event-free (EF) accounted for 25 (17.1%), 43 (29.5%), and 78 (53.4%) cases in the study, respectively. The time points for each sample were similar for the three groups. In contrast to demographic characteristics and blood counts (Supplementary Table 2), more positive culture results and antibiotics were observed in the PI group.

### Alternation of the fecal microbiome in pulmonary disorders

**Fig. 2:**
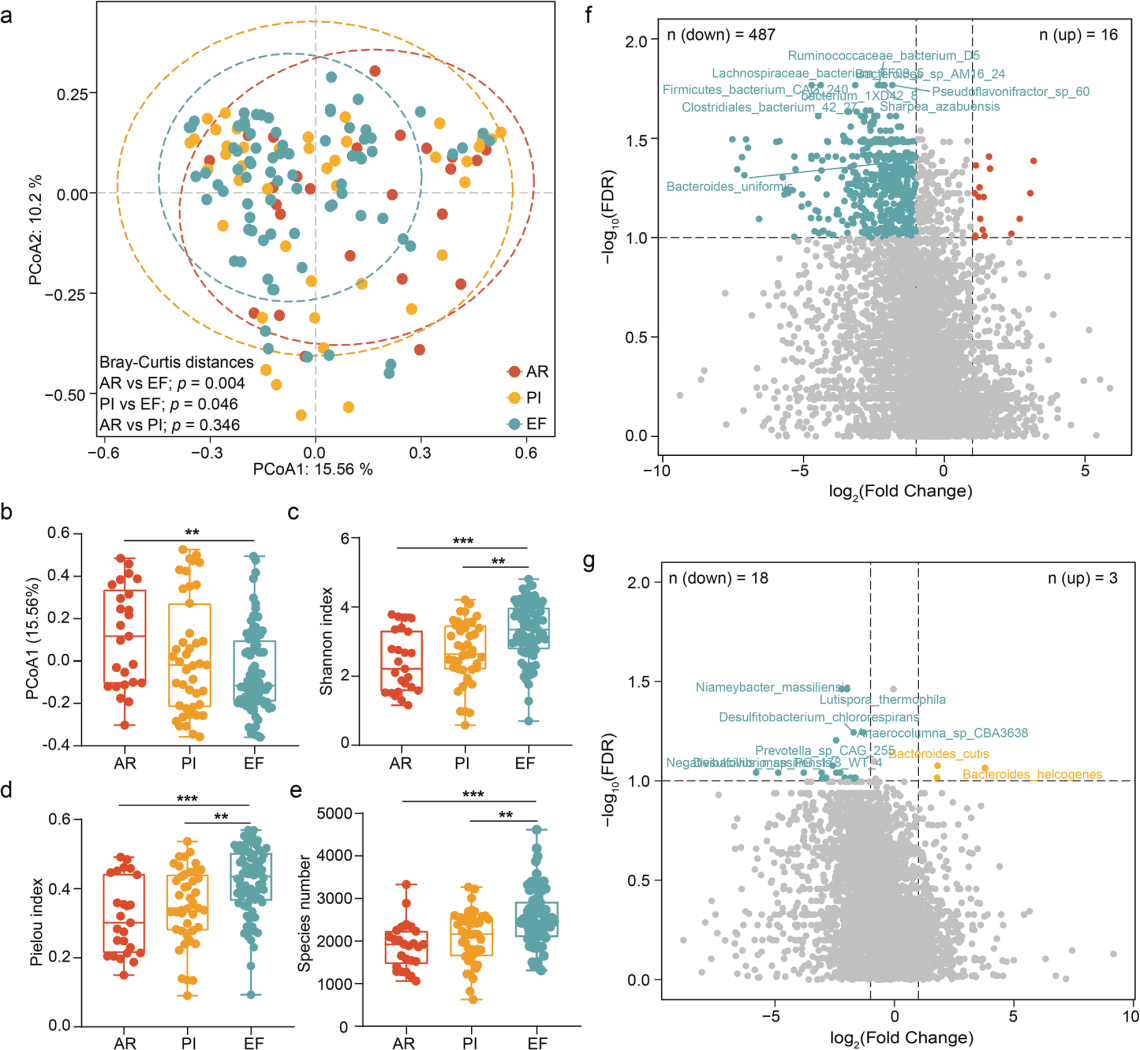
**a** Principal co-ordinates analysis (PCoA) of species across three groups. The PERMANOVA was used to define significant differences across allograft rejection (AR), pulmonary infection (PI), and event-free (EF). **b**–**e** The most principal co-ordinates, Shannon index, Pielou index, and species number of gut microbiotas in AR, PI, and EF. The center line of the box represents the median, and the box bounds represents the inter-quartile range. The whiskers span 1.5-fold the inter-quartile range. The comparison among the groups was tested by two-sided Kruskal–Wallis test, **p < 0.01 and ***p < 0.001. **f** The significantly differential species in **f** AR vs EF and **g** PI vs EF. The significantly differential species were defined by false discovery rate less than 0.1 in Wilcox Rank-Sum Test, and fold change between two groups more than 2. The red, yellow and green dots represent AR, PI, and EF, respectively

*Firmicutes* and *Bacteroidetes* followed by *Proteobacteria* and *Actinobacteria* were the most abundant phyla in the LTRs, consistent with previous cohorts from healthy individuals (Fig. 1b and Supplementary Table 3). And 32 genera were shared by ≥75% of the samples among the four most abundant phyla (Fig. 1c). The prevalence of *Bacteroidetes* phyla significantly decreased in AR, compared with EF (*p* = 0.043, Supplementary Fig. 2). The score plots of the Principal Co-ordinates Analysis (PCoA) at the species level showed a detectable alteration in the overall structure of the gut microbiomes of the AR/PI compared to EF. (Fig. 2a, b, Permanova test based on Bray-Curtis distances, AR vs EF, *p* = 0.004; PI vs EF, *p* = 0.046). Furthermore, a significant microbial gene depletion was observed in the individuals with AR and PI (PCoA1, Shannon index, Pielou index, and Species number, Fig. 2c–e).

**Fig. 3:**
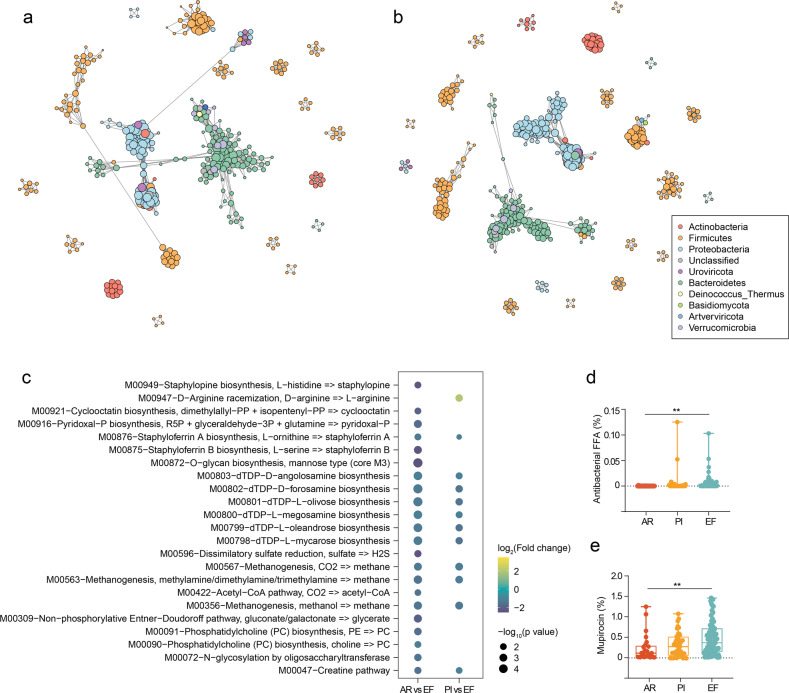
The microbial association network for **a** allograft disorder and **b** EF specimens. The co-abundance correlation between the species was calculated using SparCC correlation. All significant correlations with BH-adjusted *p* < 0.05 were included. Each node represents a microbial species. Edges between nodes represent correlations. **c** The significant changed Kyoto Encyclopedia of Genes and Genomes (KEGG) modules in AR vs EF and PI vs EF. The significantly differential modules were defined by false discovery rate less than 0.1 in Wilcox Rank-Sum Test and fold change between two groups more than 2. **d** Antibacterial FFA and **e** mupirocin-related genes in gut microbiota of three groups. The center line of the box represents the median, and the box bounds represents the inter-quartile range. The whiskers span 1.5-fold the inter-quartile range. The comparison among the groups was tested by Kruskal–Wallis test. **p < 0.01

In total, 487 species were depleted, and 16 were enriched in the AR specimens compared to EF (Fig. 2f and Supplementary Table 4, False discovery rate (FDR) < 0.1 and >2-fold change (FC)). These results are similar with previously reported findings, such as a relative depletion in probiotics bacteria (like *Bacteroides uniformis*, *Lachnospiraceae bacterium*, *Blautia obeum*, and *Phascolarctobacterium succinatutens*), and enrichment of *Enterococcus phage_IME_EFm1* and *Desulfovibrio sp_An276* in patients with pulmonary inflammation. In these species, bacteria families from *Bacteroides* and *Lachnospiraceae* are known producers of short-chain fatty acids (particularly butyrate), which play crucial roles in boosting host immunity.^20,21^ Moreover, three species were significantly enriched and 18 were decreased in the PI samples (Fig. 2g and Supplementary Table 5). However, the differences between the AR and PI groups were not be observed at the species level (Supplementary Fig. 3). Next, we combined the AR and PI samples into an allograft disorder group. The species shared by more than 50% of the subjects in the allograft disorder or EF group were used to construct a microbial co-abundance network. The allograft disorder and EF group differed significantly in the characteristics of the gut microbial network. Observably, the gut microbiota of the EF group formed a more robust community (Fig. 3a, b). Fewer edge numbers, node numbers, net connectivity, and net vulnerability were observed in the network of allograft disorder samples than EF, indicating higher network connectivity in the EF LTRs (Supplementary Table 6). We asked whether any key species drive the differences between the networks based on the parameters of average degree, closeness centrality, and betweenness centrality. We identified a bacterial species of the *Bacteroides* family, indexed uniformis, which enriched EF (Relative abundance = 2.4%) and significantly reduced in AR (Relative abundance = 0.6%, FDR = 0.04). It was reported as an immunomodulatory probiotic, optimizing the systemic Th1/Th2 balance to reduce autoimmune disorders in experimental models.^22^ Furthermore, Netshift analysis revealed that the *Bacteroides uniformis* had a higher neighbor shift (NESH) core (1.125) and stronger betweenness among 39 driven species (Supplementary Table 7). This finding supports that the *Bacteroides uniformis* is the main driver species for the changed microbial community.

We further investigated the function of the altered gut microbiome. Kyoto Encyclopedia of Genes and Genomes (KEGG) module-based analysis showed that mannose biosynthesis, N-glycosylation by oligosaccharyltransferase and methane metabolism were significantly disturbed in the AR samples (Fig. 3c and Supplementary Table 8). The mannose supplementation directly suppressed macrophage TNF-α production by reducing the glyceraldehyde 3-phosphate level in the gut.^23^ Moreover, the increased inositol phosphate metabolism and ergocalciferol biosynthesis were observed in the PI specimens (Supplementary Table 9). The correlation analysis showed that reduced metabolism positively associated with lower probiotics (Supplementary Table 10). In addition, an antibiotic resistance gene analysis showed that more antibacterial-free fatty acids and mupirocin-related species were observed in the EF specimens (Fig. 3d, e). Our data revealed that dysfunctional oligomer metabolism in gut microbiome functionality results from probiotics decline.

### Alternation of fecal microbiome correlates with systemic immune status

**Fig. 4:**
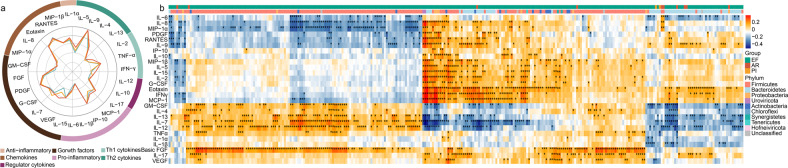
**a** Radar plot shows the log_10_-median expression of 27 cytokines in the serum across three groups. Circular distribution of cytokines is color-coded by seven functional categories and ticks show in increase in expression from the inside to the outside of the circle. **b** The correlation of the selected fecal microbiome with serum cytokines. Species with enrichment in either AR, PI, or EF samples indicated by the colored bar along the top of the heatmap. Black stars within heatmap boxes indicate significant results. *p < 0.05

Based on the observation that the gut microbiota is significantly altered in AR and PI, we hypothesized that these compositional changes play a role in exacerbating disease by contributing to dysregulation of the immune response. To investigate alterations in the immune profiles after lung transplantation, the serum levels of 27 cytokines, including seven functional categories, were compared across the three groups. The detected cytokines are involved in tissue remodeling, immunoregulation, inflammation, and so on. Their patterns differed significantly across the three groups (Fig. 4a and Supplementary Table 11). The AR and PI patients showed significant inflammatory stress with 12 cytokines, such as IL-6, IL-12, IL-17 and TNF-α, significantly up-regulated. Subsequently, we found that the gut microbiota was significantly associated with the up-regulated cytokines in the correlation analysis. The serum IL-6, identified as a key factor in the graft rejection, positively correlated with increased *Enterococcus* spp. and *Lactococcus* spp. in AR patients (Fig. 4b and Supplementary Table 12). In addition, the reduction of 16 *Bacteroides* and 7 *Clostridium* species was negatively correlated with IL-6. Interestingly, we found 3 *Enterococcus phage* species, which were enriched in EF and reported to reduce the pathogenic bacteria with minimal damage to the normal flora, were negatively associated with several inflammatory cytokines (MIP-1β, IL-1α, TNFα and IL-17). In addition, the *Bacteroides uniformis* was negatively associated with IL-1β and IL-12, which indicated that the gut microbiome is involved in allograft diseases possibly via modulating host immune responses in lung transplant.^24,25^

### Alternation of fecal metagenome correlates with serum metabolites

**Fig. 5:**
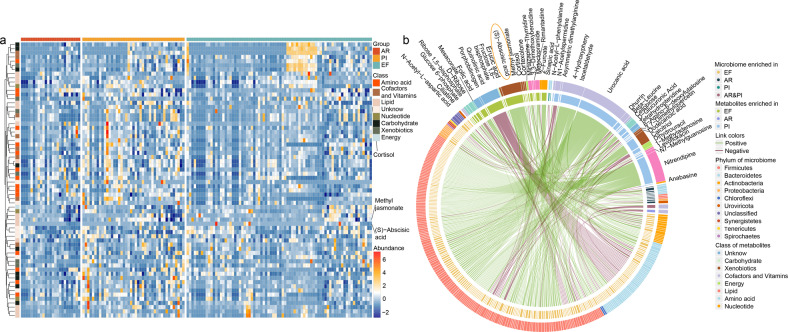
**a** The 52 significant altered metabolites among AR, PI, and EF. The comparison between two groups was tested by the Student’s *t* test. **b** The correlation of the selected fecal microbiome with serum metabolites. The significantly correlated (calculated by Spearman correlation, *p* < 0.01) species and metabolite are showed in the cycle. Species and metabolites with enrichment in either AR, PI, or EF samples are indicated by the colored bar of the inside track. The phylum of the microbiome and class metabolites are indicated by the colored bar of outside the track. The green and yellow lines represent positive and negative correlation, respectively

Next, we asked which environmental factors explain the levels of the altered gut microbiome. Serum metabolites are known to play a key role in mediating the metabolic and immune interactions between the microbiome and its host, thus providing a fundamental view into the complex dynamics of environmental exposures. Consistent with the gut microbiome, AR individuals exhibited a broad set of perturbations with a major depletion pattern in serum metabolite levels. Fifty-two altered metabolites were identified among the three groups (Fig. 5a and Supplementary Tables 13 and 14). Several depleted metabolites, such as glucose-6-phosphate, were previously reported to attenuate lung injury.^26^ Furthermore, the correlation between the significantly changed gut microbial species and serum metabolites was analyzed. We found that lowering lipid ((S)-abscisic acid, methyl jasmonate and cortisol) positively correlated with these decreasing probiotics (Fig. 5b and Supplementary Table 15). These species are mainly derived from *Bacteroidetes* (28 species) and *Clostridium* (20 species). Of these, (S)-abscisic acid, which was reported to reduce lung injury through peroxisome proliferator activated receptor γ (PPAR-γ) signaling, is strongly associated with the deletion of 86 species.^27^ In addition, the loss of *Bacteroides uniformis* significant associated with increased quinolinic acid which is well-known neurotoxin. These results suggest that the gut probiotics, play a potential protective role in AR development, mediated by an array of circulating blood metabolites, several of which were previously shown to play a central role in pulmonary inflammation, while others were not reported. Thus, upon further validation in experimental studies, these metabolites may form new targets to attenuate disease risk.

### A machine learning classifier for pulmonary disorders

**Fig. 6:**
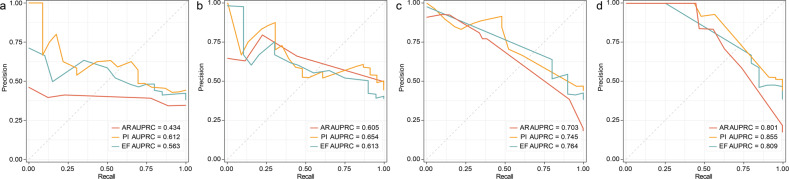
The precision-recall curves (PRC) of support vector machine (SVM) models based on the features from **a** clinical data, **b** serum metabolites, **c** gut microbiota, and **d** multi-omics data. The red, yellow, and green lines represent the diagnosis of AR, PI, and EF, respectively

The accurate diagnosis of AR and PI is important for clinical practice. We hypothesized that the distinct microbial, and metabolic signature could predict the AR and PI in lung transplant patients. At first, 146 specimens were subdivided in to a training cohort (104 samples) and multi-regional validation cohort (42 samples, Supplementary Table 16). Based on a support vector machine (SVM) approach, we constructed machine learning models by employing the clinical parameters, including blood counts and serum cytokines, and used the significantly changed species of the gut microbiome and the significantly disturbed serum metabolites to distinguish AR, PI, or EF from the subjects (Fig. 6a–d). When identifying AR, PI, or EF, the precision-recall curve (PRC) showed that the predicted power of the SVM models based on the clinical parameters was significantly lower than models using microbiome and metabolome data in the validated samples. However, the single microbiome (area under the precision–recall curve, AUPRC range = 0.703–0.764) and metabolome (AUPRC range = 0.605–0.654) were also less effective. The SVM models based on the multi-omics data (integration of clinical, microbial and metabolic features) accurately predicted AR, PI, or EF in the validated subjects (AUPRC = 0.801 for AR, AUPRC = 0.855 for PI, and AUPRC = 0.809 for EF). These data supported a high predictive power of the multi-omics for diagnosis of AR and PI in lung transplant.

## Discussion

In this prospective, observational, multi-center study, we obtained a comprehensive multi-omics profiling from 146 samples of LTRs. To our knowledge, this is the first clinical cohort of LTRs assembled for this type of analysis. By comparing the gut microbiome profiles of AR and PI with EF controls, we found a unique signature of AR, with hundreds of significantly reduced microbiomes. Besides the loss of gut microbial diversity, the specific functions, such as mannose biosynthesis, were disrupted. *Bacteroides uniformis* was identified as the keystone species for the constructing the microbial network. Meanwhile, several altered intestinal species, including *Bacteroides uniformis* and *Enterococcus phage*, were significantly correlated with the systemic immune status and metabolites. Based on the SVM approach, we proposed and validated an effective multi-omics classifier in discriminating AR and PI patients.

In various clinical cohorts with organ transplantation, patients succumb due to the loss of “health-promoting” species and the overgrowth of “disease-promoting” species.^28^ Haak et al. reported that the absent representation of butyrate-producing bacteria in the fecal microbiota was associated with increased susceptibility to respiratory infection in allogeneic hematopoietic stem cells and kidney transplant recipients.^29^ In addition, gut microbiota dysbiosis affects the clinical outcome of various of organ transplantations. Kato et al. showed that intestinal *Bacteroides* and *Streptococcus* were increased while *Enterococcus* and *Clostridium* were significantly decreased in liver transplant recipients with AR compared to healthy recipients.^30^ Moreover, *Lactobacillales* and *Enterobacteriales* are negatively correlated with AR status in patients with intestinal transplants.^31^ However, previous studies on the gut microbiome in solid organ transplantation have been constrained by 16S ribosomal RNA (rRNA), which provides only limited resolution. Similar with our results, Swarte et al. proved that gut dysbiosis, including lower microbial diversity, increased abundance of unhealthy microbial species, and decreased abundance of important metabolic pathways could be observed in the both liver and kidney transplant recipients based on the shotgun metagenomics data of 1370 fecal samples.^12^

Emerging microbiome studies in pulmonary diseases, such as acute respiratory distress syndrome (ARDS) and lung cancer, have demonstrated marked abnormalities compared to healthy controls in the lower respiratory tract and gut.^32–35^ Dickson et al. analyzed bacterial communities from the bronchoalveolar lavage fluid (BALF) of ARDS patients and found that gut-associated *Bacteroides*, which were absent in the healthy controls, were detectable in 41% of the samples.^32^ Further studies confirmed that the bacterial community is characterized by predominant *Enterobacteriaceae* and had a significantly decreases diversity in the lungs of ARDS.^36^ The gut-derived *Enterobacteriaceae* and lower diversity are also highly correlated with clinical outcomes.^37^ All the existing studies have supported the existence of the “gut–lung axis.” Nevertheless, the mechanisms by which the gut microbiota affects the immune responses in the lungs remain undetermined.

The crosstalk between microorganisms and the host is complex, and our current understanding of these interactions is in its infancy. Our study showed that the loss of *Bacteroides uniformis* negatively correlates with IL-1β and IL-12, and the increased two cytokines could modulate serious allograft dysfunction.^13,24^ Notably, the administration of *Bacteroides uniformis* was reported to attenuate systemic and adipose tissue inflammation, inducing changes in the host immunity.^22^ Fabersani et al. showed that an increased concentration of the anti-inflammatory cytokine IL-10 was involved in Treg induction and type 2 innate lymphoid cell activation. Therefore, the research on the mechanism underlying the *Bacteroides uniformis*’ modulation on IL-1β and IL-12 is worth further investigation in the LTRs. In addition, we also found that the richness of the mannose-producing microbiome, is associated with the reduction of graft-versus-host disease. Further analysis emphasized that the “lung–gut” axial might be built by the systematic alteration of metabolites and cytokines caused by the dysbiosis microbial community.

The International Society for Heart and Lung Transplantation (ISHLT) reported that severe infection (33.1%) and graft failure (16.1%) were the leading causes of death within 1-year after lung transplant.^4^ The early clinical symptoms of PI and AR, including chest CT and blood counts, are similar, and treatments for severe infection and rejection are mutually exclusive. Several studies have shown an early and accurate diagnosis of AR and PI is crucial in clinical practices, which might enable treatment before irreversible organ function damage. Various biomarkers of AR were identified, such as plasma cell-free DNA and microRNAs (area under curve (AUC) = 0.72–0.89).^38,39^ However, there are no independent validation cohorts in these researches. Despite the relatively small samples used to build the SVM model, the diagnostic power of the SVM classifier for the predicted and objective outcomes was highly significant. More importantly, we validated this multi-omics model in an independent external cohort. Further analysis of the multi-omics model contribution showed that *Bacterium 1XD428* and *Bacteroides uniformis* contributed the most to the detection of AR (Supplementary Table 17). In addition, *Niameybacter massiliensis* and *Bacteroides cutis* had the largest contribution for the detection of PI (Supplementary Table 18). These findings further showed that *Bacteroides uniformis* might play a modifying role in the pathogenesis of AR.

The present study had some limitations that warrant further exploration. First, the recognized variation in the gut microbiome data between the individuals and confounders, such as medication status and indications for lung transplantation, likely limited our analysis to detect additional significant taxonomic and functional biomarkers for AR and PI in the LTRs. Further experimental studies are need to verify our findings and investigate the underlying mechanism. Second, the follow-up time was relatively short, and the association between the gut microbiota and CLAD was not investigated in this study. A longitudinal analysis with a long-term follow-up of patients with AR, PI, and CLAD would be interesting if paired with samples collected from of the lower respiratory tract and blood to evaluate the potential effect from the gut.

Using a comprehensive gut microbiome, serum cytokine, and metabolomic profiling, we present a deep mapping of the LTRs. Our analysis unraveled new paradigms and therapeutic directions, for example the depletion of *Bacteroides uniformis* in AR. It may form the basis for future mechanistic experiments, preclinical and human interventional studies.

## Material and methods

### Study design and participants

We prospectively established a cohort (ChiCTR1900028066) of LTRs at Shanghai Pulmonary Hospital (SPH), The First Affiliated Hospital of Guangzhou Medical University, Changhai Hospital, and Sun Yat-sen Memorial Hospital from May 2020. Our study aimed to analyze the clinical significance of intestinal microbiome characteristics in LTRs. Due to the severe acute rejections and infections that mainly occur within 1-year after lung transplant, we limited all the enrolled samples in this period. AR was diagnosed and graded according to the ISHLT guideline.^40^ The diagnosis of PI was confirmed by a multidisciplinary team, including thoracic surgeons, respiratory physicians, pathologists, and radiologists. The cases without any symptoms and negative pathological based on the findings with most recent biopsies were considered EF samples. All the fecal samples and paired blood specimens were collected under strict sterile conditions during hospitalization for surveillance bronchoscopy or treatment. Specimens from inpatients were taken within 12 hours of admission and stored at −80 °C until used. Samples taken from the same patient at least two months apart could be included in analysis. This study achieved the approval from the Institutional Review Board of SPH (IRB number: K19-164), and written informed consent was obtained from all enrolled LTRs. The specimens from the perioperative period were also excluded due to the irregular diet. The specific treatment regimens and details of the specimen collection are detailed in the Supplementary Material.

### Metagenomic sequencing of feces

Consistent with our previous study, genomic DNA was extracted from the fecal specimens via the QIAamp PowerFecal DNA kit (#51804, QIAGEN, USA), and the sequencing library for each sample was prepared using the KAPA HyperPlus Library Preparation Kit (#KK8514, Roche).^41^ Shotgun sequencing was performed on the Illumina Novaseq 6000 platform at Adfontes Biotechnology Co. (Shanghai, China) to obtain 150 bp forward and reverse paired-end reads. On average, each sample yielded more than 8 G of raw data.

### Serum untargeted metabolomics

Frozen serum samples were thawed at 4 °C. The LC-MS analysis was performed using an Orbitrap Exploris 120 (Thermo Fisher Scientific, USA) and a Vanquish UPLC System (Thermo Fisher Scientific, USA) for an Orbitrap Q Exactive mass spectrometer for untargeted metabolomics detection. A detailed description of the metabolomics extraction, data processing, and analysis is detailed in the Supplementary Material.

### Multiplex immunoassay analysis of cytokines

Thawed serum samples were diluted twofold and centrifuged at 3000 × *g* for 5 min. A multiplex immunoassay of 27 cytokines (Bio-Rad Laboratories Inc, Hercules, CA) was used to analyze the cytokines and chemokines in the serum. The analytes were measured using the Luminex X-200 system (Luminex Corp, Austin, TX). An 8-point standard curve in duplicate, was included on every 96-well plate. Results with more than a half of the samples above the limit of detection were selected for further analysis.

### Bioinformatics analysis and statistical tests

#### Microbiota data

The raw sequencing reads were preprocessed using Trimmomatic v0.39 to remove the low-quality reads.^42^ Bowtie2.4.1 software was used to filter out the redundant originating from the host. Paired-end reads with high quality from each sample were de novo assembled into contigs of at least 500 bp by SOAPdenovo software v2.04 (http://soap.genomics.org.cn/soapdenovo.html). Genes were predicted by MetaGeneMark (v2.10, http://topaz.gatech.edu/GeneMark/). CD-HIT (v4.8.1, http://www.bioinformatics.org/cd-hit) was used to construct the non-redundant gene catalog for the microbial genes.^43^ High-quality reads were mapped onto the gene catalog via SOAP2 software (v2.21, http://soap.genomics.org.cn/), and the counts of the reads that aligned to a gene were normalized by gene length to calculate the abundance of each gene in an individual sample.^44^ The taxonomy annotation of the non-redundant gene catalog was performed using DIAMOND software (v0.9.9.110, https://github.com/bbuchfink/diamond/) based on the NCBI NR databases (Version 2018-01-02, https://www.ncbi.nlm.nih.gov/).^45^ The functional annotation of the non-redundant gene catalog was done using DIAMOND software based on the KEGG database (v2019.10, http://www.kegg.jp/kegg/).

For the alpha-diversity and beta-diversity analysis of the gut microbiota, the Shannon index, observed species, Pielou index, and PCoA based on species were performed using the R vegan package. The Wilcox Rank-Sum Test of single variation was used to determine the species and functional features significantly differentiated between the groups, and the differences were defined as significant differences when the FDR was <0.1 and >2-FC.

The AR and PI were combined into an allograft disorders group. Then, the species that were shared in more than 50% of the samples were used to construct a microbial association network for the allograft disorders or EF group using FastSpar software based on the SparCC algorithm. The *p* values for the correlation between two species were calculated from 1000 bootstraps and adjusted by the Original FDR method of Benjamini and Hochberg to obtain the significance of the correlations. The network attributes, such as the node (vertex) number, edge (link) number, mean degree, density, connectivity, and average path, were calculated using the R package graph. The driven species were identified by NetShift from altered microbiomes.^46^ Altered species from allograft disorders and EF group were defined as the case and control, respectively. Then, the betweenness value each selected species was obtained by a Spearman’s rank correlation analysis. The betweenness values of selected species were input into the NetShift package to calculate NESH cores.

#### Analysis of metabolomic data

At first, the qualified raw data were converted to mzXML format by MSConvert in the ProteoWizard software package (v3.0.8789).^47^ The XCMS software packages were used for feature detection, retention time correction, and alignment.^48^ We identified metabolites by accuracy mass (<20 ppm) and the MS/MS data and matched these with HMDB, METLIN, MAASSBANK, LipidMaps, mzcloud, and KEGG. The robust LOESS signal correction (QC-RLSC) was applied for data normalization to correct for any systematic bias. After normalization, only ion peaks with relative standard deviation less than 30% in the QC were kept, ensuring proper metabolite identification. A Student’s *t* test was performed to examine the overall distribution of the serum metabolites between the groups, with a threshold of a *p* value < 0.05 for considering the significant differential metabolites. The significant differential metabolites were visualized by the R Heatmap package. We subjected the differential metabolites to a pathway analysis based on MetaboAnalys.^49^

### Multiplex immunoassay data analysis

For the radar chart visualization, the log_10_-median expression values of each factor were calculated for each group. The highest log_10_ expression value from all the factors was arbitrarily set to 1 and plotted as the maximum value in the corresponding figures using the R fmsb package. We used the Wilcox Rank-Sum Test to determine the immune factors that were significantly differentiated between the groups.

### Multi-omics association analysis

A multi-omics analysis based on the intestinal microbiota and the serum metabolites and cytokine data was carried out. First, we selected key species from the significantly different species (AR vs EF and PI vs EF). Then, using the association between the gut microbiota, serum metabolites, and cytokines in the LTRs, we constructed a correlation analysis using Spearman’s correlations in R version 4.1 (psych package). The differences were defined as significant when the *p* value was <0.05. The correlations were visualized by the R Heatmap and cyclize package.

### Machine learning model construction

The SVM algorithm was utilized to construct one-versus-rest for multi-class classification by scikit-learn (Python, https://github.com/j-bac/scikit-dimension).^50^ The target outcome was a diagnosis of AR from PI and EF, PI from AR and EF, and EF from AR and PI. The clinical parameters included blood counts and serum cytokines, and the significantly changed species in the gut microbiome (*p* < 0.05 and >2-FC) or the significantly changed serum metabolites (*p* < 0.05) were used to construct SVM models. Integration of all the above variables was also used to construct the SVM model. We assigned the 104 samples of SPH to the training cohort and 42 samples of the other three centers to a validated cohort. We performed the cross-validation and parameter optimization was used to develop the models in the training samples. The validated samples were used for testing. A PRC was determined, and the AUPRC was calculated to evaluate the diagnostic values of the classifiers. We identify Feature contribution index were obtained by identifying the best alpha value, fitting a final model on the whole data set, and reporting features contribution of this model.

### Supplementary information

Supplementary Materials

Supplementary Tables

## Data Availability

The metagenomic and metabolomic raw data generated in this study have been deposited in the Genome Sequence Archive (GSA) database of the National Genomics Data Center (NGDC, https://bigd.big.ac.cn/) under the accession number: PRJCA016873.
