# Supplementary material for: Intestinal microbiota links to allograft stability after lung transplantation: a prospective cohort study
Source: Signal Transduct Target Ther. 2023 Sep 1;8:326. doi: 10.1038/s41392-023-01515-3 (PMC10471611; doi:10.1038/s41392-023-01515-3)
Supplement: Supplementary file 1 — Supplementary Materials [file 41392_2023_1515_MOESM1_ESM.docx]

Supplementary Materials for

Intestinal microbiota links to allograft stability after lung transplantation: a prospective cohort study

Junqi Wu, Chongwu Li, Peigen Gao, Chenhong Zhang, Pei Zhang, Lei Zhang, Chenyang Dai, Kunpeng Zhang, Bowen Shi, Mengyang Liu, Junmeng Zheng, Bo Pan, Zhan Chen, Chao Zhang, Wanqing Liao, Weihua Pan, Wenjie Fang, Chang Chen

Correspondence to: Weihua Pan ([panweihua9@sina.com](mailto:panweihua9@sina.com)); Wenjie Fang ([fangwenjie1990@126.com](mailto:fangwenjie1990@126.com)); Chang Chen ([chenthoracic@163.com](mailto:chenthoracic@163.com))

**This PDF file includes:**

Materials and Methods

Supplementary Figs. 1 to 3

Reference

Materials and Methods

**Diagnosis of pulmonary diseases**

Allograft rejection (AR) presents with nonspecific features, such as shortness of breath, cough with or without sputum production and even low-grade fever. According to the guideline of The International Society for Heart and Lung Transplantation (ISHLT), pathological findings in transbronchial biopsy specimens are the gold standard for the diagnosis of AR after lung transplant^1^. The diagnosis of acute rejections s, Grade A0 (none), Grade A1 (minimal), Grade A2 (mild), Grade A3 (moderate) and Grade A4 (severe), are based on perivascular and interstitial mononuclear infiltrate. In addition, the small airways inflammation, lymphocytic bronchiolitis, Grade B0 (none), Grade B1 (low grade), Grade B2 (high grade) and BX (ungradable) are also included. The chronic airway rejection is excluded due to the lower incidence during the first year after transplant.

The diagnostic criteria for pulmonary infection (PI) are based on clinical symptoms, laboratory parameters, blood gas analysis, lung function tests, imaging studies, and bronchoscopy, and are summarized as follows^2,3^:

(1) Clinical symptoms: at least one of the following: (a) fever 38°C or hypothermia, 36.5°C with no other recognized source; (b) leukocyte count, 4000 or 15000/mm^3^; (c) purulent secretions; (d) new onset or worsening cough, dyspnea, tachypnea or plural rub, rales, or bronchial breath sounds; (e) worsening gas exchange (O_2_ desaturation, PaO_2_/FiO_2_, 240) increasing the O_2_ requirement and the ventilation demand; and (f) pleural effusion.

(2) Radiology: A new or progressive alveolar or interstitial infiltrate or cavitation on chest X-ray or CT-scan that could not be explained by any other noninfectious cause. (3) Microbiology: at least one of the following: (a) isolation or detection of any microorganism in blood unrelated to other sources; (b) isolation or detection of any microorganisms in representative respiratory samples including sputum, bronchial secretions, BALF, bronchial sterile brushing or pleural fluid; and (c) 5% of BALF-obtained cells contained intracellular bacteria on direct microscopic examination.

As a supplement of traditional microbiological testing platform, other microbiological tools such as metagenomic next generation sequencing (mNGS) may also help in the diagnosis of infection. It is noteworthy that positive bacterial culture could be due to the presence of respiratory pathogens or colonized bacteria. In addition, if fungal infection was suspected, other tests such as galactomannan, 1,3-b-D-glucan assay or even PCR, could be performed. The diagnosis of some other infection, such as L. pneumophila, Mycoplasma pneumoniae, Chlamydia pneumoniae, Chlamydia psittaci and influenza virus pneumonia could also be established by a 4-fold or greater rise in the IgG titer^4^. In patients with a positive culture, those who did not fulfill the clinical criteria for respiratory infection (lack of symptoms/signs and radiologic changes) were classified by 2 clinicians as colonized patients.

Event-free (EF) recipients are either discharged to common ward from the ICU or hospitalized for reexamination and were defined as having neither infection nor rejection. Each sample is assigned a diagnosis of AR, PI or EF independently by two experienced clinicians (Dr. Lei Zhang and Dr. Kunpeng Zhang). In the case of disagreement, a third clinician was consulted (Dr. Chang Chen).

**Untargeted metabolomics profile assessment**

**Sample Preparation**

Human serums were thawed at 4 ℃ and 100 μL of each sample was transfered into a 2mL centrifuge tubes with 400 μL methanol (-20 ℃) in it. Metabolites were extracted after vortexing and centrifuging, and the resulting supernatants were tranfered into another 2 mL centrifuge tube and concentrated to dry in vacuum. Samples were dissolved with 150 μL 2-chlorobenzalanine (4 ppm) 80% methanol solution and were filtered through 0.22 μm membrance for LC-MS.

**LC-MS conditions**

The LC-MS analysis was performed on an Orbitrap Exploris 120 (Thermo Fisher Scientific, USA) coupled with a Vanquish UPLC System (Thermo Fisher Scientific, USA). Chromatographic separation was carried out with an ACQUITY UPLC® HSS T3 column (150×2.1 mm, 1.8 µm) (Waters, Milford, USA) maintained at 40 ℃. The temperature of the autosampler was 8 ℃. Gradient elution of analytes was carried out with 0.1% formic acid in water (C) and 0.1% formic acid in acetonitrile (D) or 5 mM ammonium formate in water (A) and acetonitrile (B) at a flow rate of 0.25 mL/min. Injection of 2 μL of each sample was done after equilibration. An increasing linear gradient of solvent B (v/v) was used as follows: 0~1 min, 2% B/D; 1~9 min, 2%~50% B/D; 9~12 min, 50%~98% B/D; 12~13.5 min, 98% B/D; 13.5~14 min, 98%~2% B/D; 14~20 min, 2% D-positive model (14~17 min, 2% B-negative model)^5^.

The ESI-MSn experiments were used with the spray voltage of 3.5 kV and -2.5 kV in positive and negative modes, respectively. Sheath gas and auxiliary gas were set at 30 and 10 arbitrary units, respectively. The capillary temperature was 325 ℃. respectively. The Orbitrap analyzer scanned over a mass range of m/z 100-1 000 for full scan at a mass resolution of 60 000. Data dependent acquisition (DDA) MS/MS experiments were performed with HCD scan. The cracking rate is 30%. Dynamic exclusion was implemented to remove some unnecessary information in MS/MS spectra^6^.

^
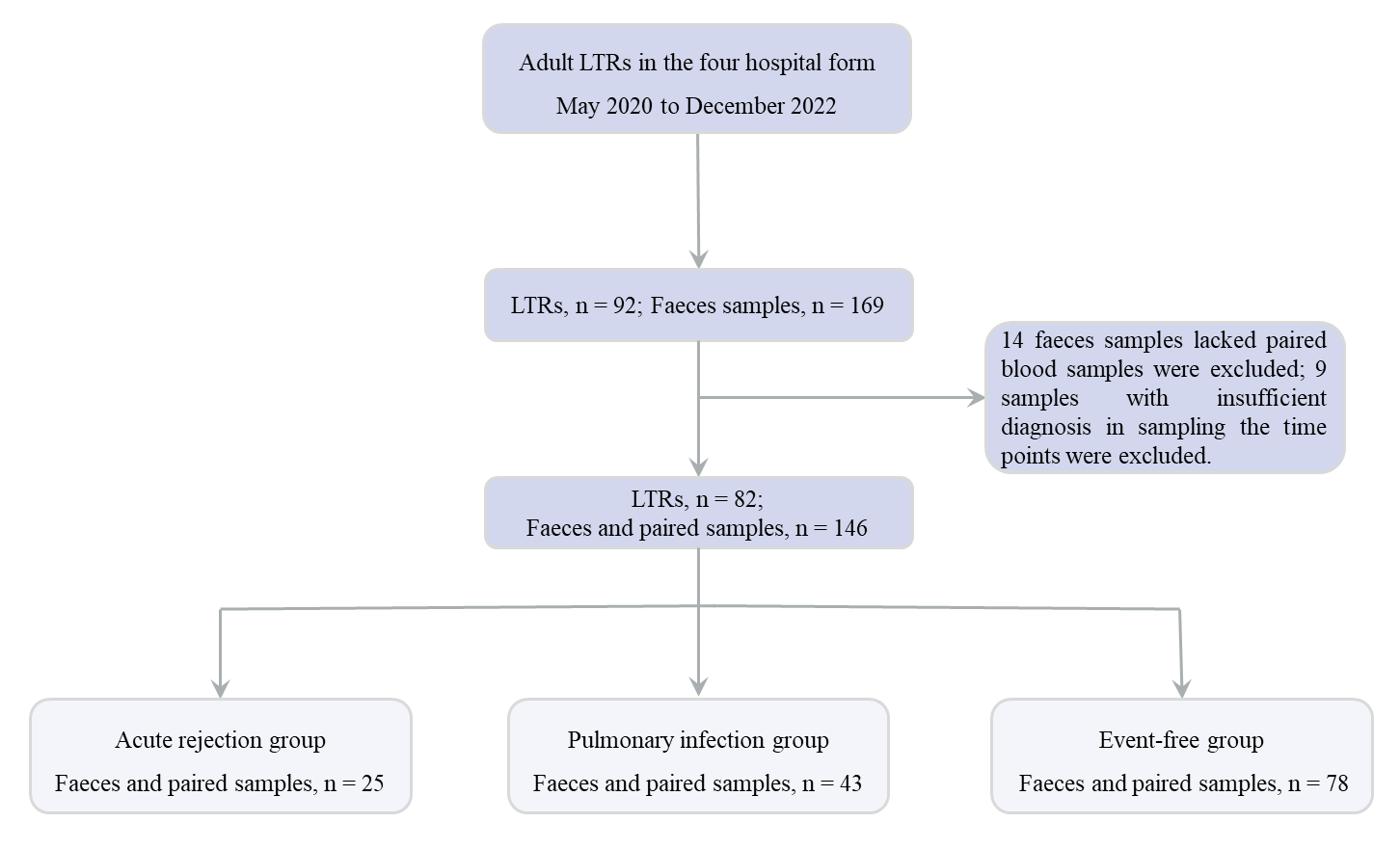
^

**Supplementary Fig. 1** Study cohort and the sample group


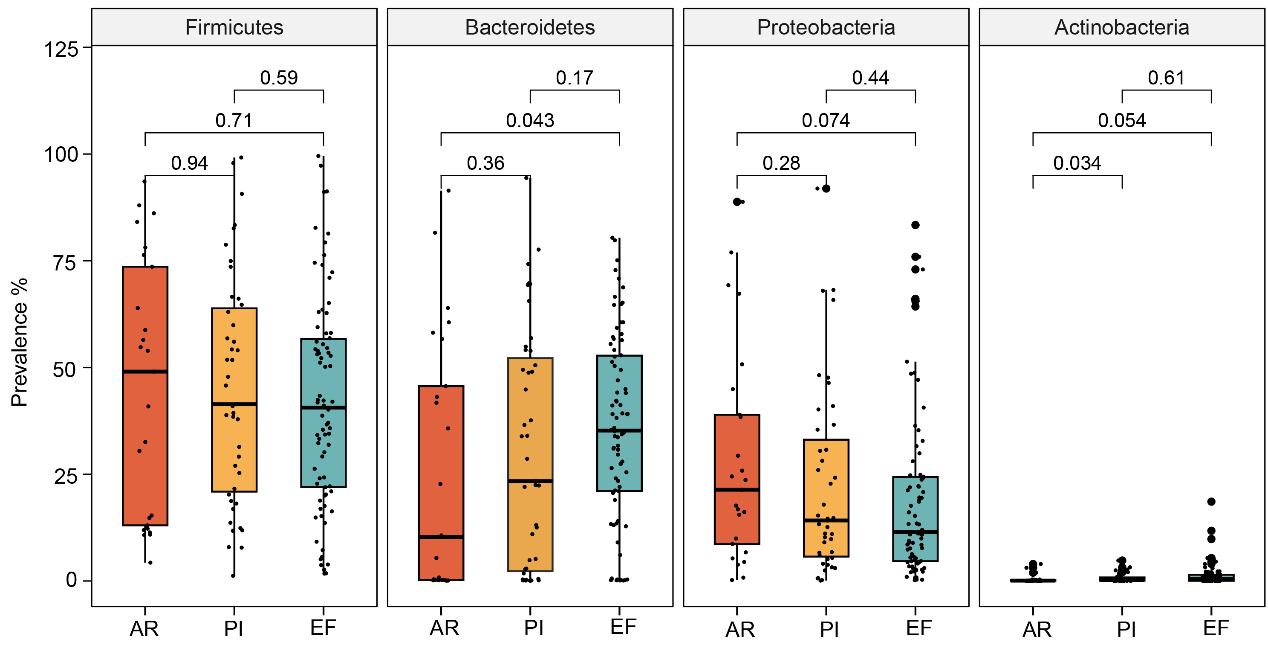


**Supplementary Fig. 2** Four most abundance across three groups (AR = Allograft rejection, PI = Pulmonary infection, EF = Event-free). The center line represents the median, and the box bounds represents the inter-quartile range. The whiskers span 1.5-fold the inter-quartile range.


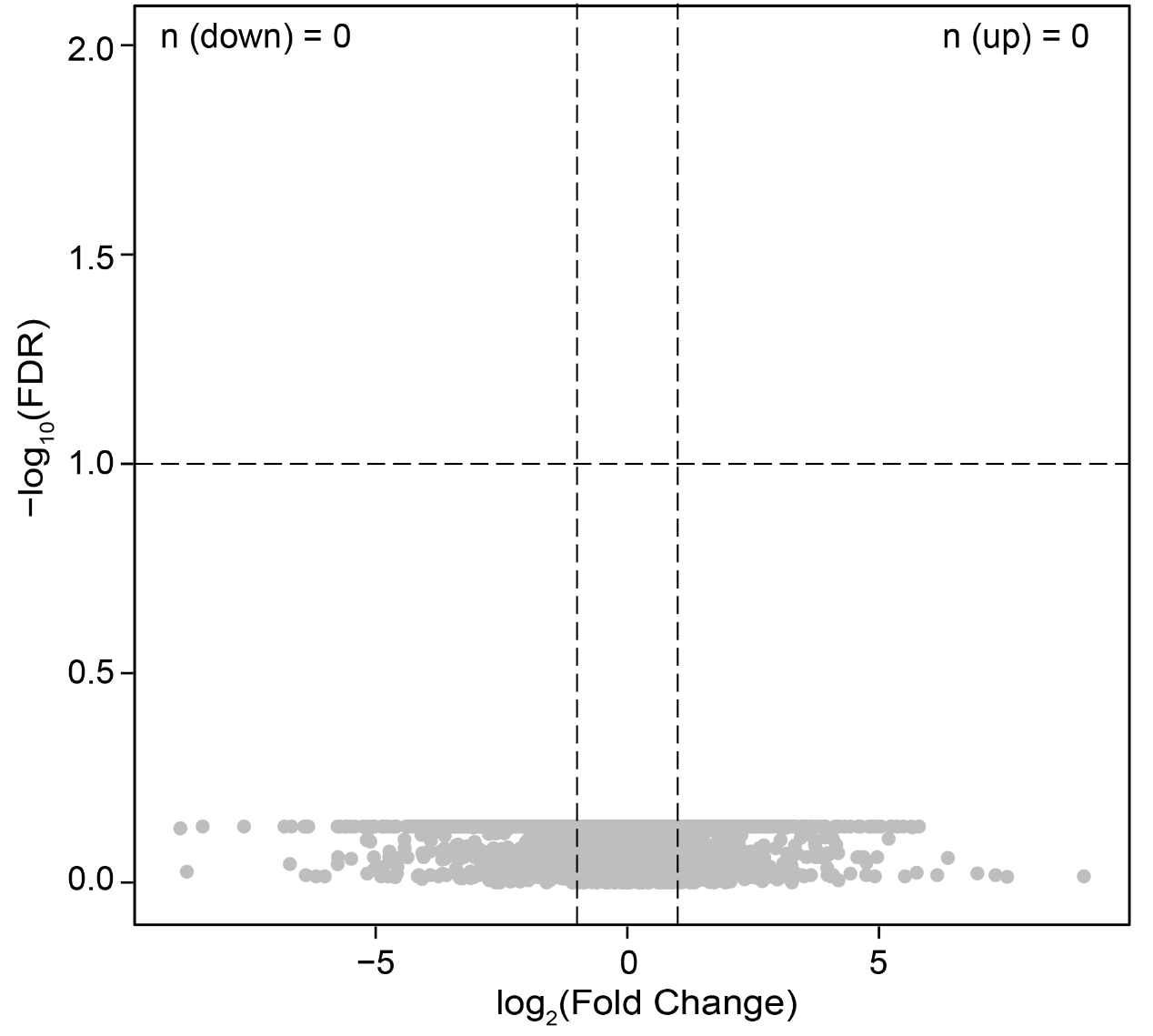


**Supplementary Fig. 3** Volcano plots of the significantly differential species in Allograft rejection (AR) vs Pulmonary infection (PI) was shown. Two-sided Wilcoxon rank-sum tests followed by Benjamini-Hochberg (BH) multiple comparison test with false discovery rate (FDR) < 0.1 and fold change > 2 or < -2. The red and purple dots represent AR and PI, respectively.

**References**

1 Stewart, S. *et al.* Revision of the 1990 working formulation for the standardization of nomenclature in the diagnosis of heart rejection. *J Heart Lung Transplant*. **24**, 1710-1720 (2005).

2 Joean, O., Welte, T. & Gottlieb, J. Chest Infections After Lung Transplantation. *Chest*. **161**, 937-948 (2022).

3 Grief, S. N. & Loza, J. K. Guidelines for the Evaluation and Treatment of Pneumonia. *Prim Care*. **45**, 485-503 (2018).

4 Aguilar-Guisado, M. *et al.* Pneumonia after lung transplantation in the RESITRA Cohort: a multicenter prospective study. *Am J Transplant*. **7**, 1989-1996 (2007).

5 Abdelhafez, O. H. *et al.* Metabolomics analysis and biological investigation of three Malvaceae plants. *Phytochem Anal*. **31**, 204-214 (2020).

6 Monnerat, G. *et al.* Aging-related compensated hypogonadism: Role of metabolomic analysis in physiopathological and therapeutic evaluation. *J Steroid Biochem Mol Biol*. **183**, 39-50 (2018).
